# Predictive Factors for Response to PD-1/PD-L1 Checkpoint Inhibition in the Field of Hepatocellular Carcinoma: Current Status and Challenges

**DOI:** 10.3390/cancers11101554

**Published:** 2019-10-14

**Authors:** Zuzana Macek Jilkova, Caroline Aspord, Thomas Decaens

**Affiliations:** 1 Université Grenoble Alpes, 38000 Grenoble, France; caroline.aspord@efs.sante.fr; 2 Institute for Advanced Biosciences, Research Center UGA/Inserm U 1209/CNRS 5309, 38700 La Tronche, France; 3 Service d’hépato-gastroentérologie, Pôle Digidune, CHU Grenoble Alpes, 38700 La Tronche, France; 4 Etablissement Français du Sang Auvergne-Rhône-Alpes, R&D-Laboratory, 38701 Grenoble, France

**Keywords:** PD-1, PD-L1, hepatocellular carcinoma, predictive factors, immunotherapy, immune checkpoint inhibition

## Abstract

Immunotherapies targeting immune checkpoints are fast-developing therapeutic approaches adopted for several tumor types that trigger unprecedented rates of durable clinical responses. Immune checkpoint programmed cell death protein 1 (PD-1), expressed primarily by T cells, and programmed cell death ligand 1 (PD-L1), expressed mainly by tumor cells, macrophages, and dendritic cells, are molecules that impede immune function, thereby allowing tumor cells to proliferate, grow and spread. PD-1/PD-L1 checkpoint inhibitors have emerged as a promising treatment strategy of hepatocellular carcinoma (HCC). However, only a minority of HCC patients benefit from this therapy. To find a niche for immune checkpoint inhibition in HCC patients, future strategies might require predictive factor-based patient selection, to identify patients who are likely to respond to the said therapy and combination strategies in order to enhance anti-tumor efficacy and clinical success. This review provides an overview of the most recent data pertaining to predictive factors for response to PD-1/PD-L1 checkpoint inhibition in the field of HCC.

## 1. PD-1/PD-L1/PD-L2: A Physiological Immune Checkpoint Axis Exploited by Cancer Cells and Viruses to Escape Immunity

Programmed cell death protein 1 (PD-1) was discovered in 1992 by the group led by Tasuku Honjo [1], who received the 2018 Nobel Prize in Physiology or Medicine for this discovery. Honjo and his group of researchers described PD-1 antigen expression on the surface of stimulated mouse T and B lymphocytes [2] and showed the importance of PD-1 activation during the late phase of immune responses, involvement in the effector phase, memory response, and chronic infections in peripheral tissues. This pathway displays a physiologic role in maintaining self-tolerance and dampening immune responses to immune reactions. Programmed cell death ligand 1 (PD-L1) was identified as PD-1 ligand by Honjo’s group in 2000, as a receptor expressed by antigen-presenting cells, primarily in the heart, lungs, kidney, and placenta [3]. In 2001, the second ligand for PD-1, i.e., PD-L2, was described, and the expression of PD-1 ligands on tumor cell lines was demonstrated [4]. This report suggested, for the first time, that blocking the PD-1 pathway might enhance anti-tumor immunity.

At present, it is known that PD-L1 is expressed in non-lymphoid and lymphoid tissues, whereas PD-L2 expression is more restricted. PD-L1 expression is upregulated upon activation in hematopoietic cells, especially antigen-presenting cells such as dendritic cells and macrophages. Most importantly, PD-L1 is expressed in different tumor cells and in virus-infected cells, and upon ligation with PD-1, it directly inhibits T-cell proliferation and T-cell effector functions such as IFN-gamma production and cytotoxic activity against the target cells [5].

## 2. PD-1/PD-L1 Pathway in Hepatocellular Carcinoma

A recent study based on tumor samples with advanced solid tumors and melanoma depicted hepatocellular carcinoma (HCC) as a tumor type with low/moderate immunogenicity [6], which may explain the lower rate of response of HCC patients to immune checkpoint blockers compared to melanoma patients.

HCC is commonly developed on the background of chronic liver disease (chronic hepatitis B virus (HBV) or hepatitis C virus (HCV) infection, metabolic disorders, or chronic alcohol consumption), which promotes an immunosuppressive status of liver and T-cell exhaustion [7,8]. During tumor development and growth, the effective anti-tumor immune surveillance in the liver microenvironment is impaired, and immune checkpoints, especially the PD-1/PD-L1 signaling pathway, are greatly involved in the said process [9]. In patients with HCC, the expression of PD-1 was increased in CD8^+^ T cells [10], and the high frequency of both circulating and tumor-infiltrating PD-1^+^ CD8^+^ T cells was associated with progression following curative hepatic resection in patients who were never treated via immunotherapy [11]. Furthermore, high PD-1 expression on tumor-infiltrating lymphocytes and the correlation between an exhausted phenotype and impaired effector function have been observed in HCC patients [12,13]. The expression of PD-L1 in HCC cells inhibits function of T cells in the liver tumor microenvironment. Not surprisingly, high PD-L1 expression on tumor cells was determined as a predictor of recurrence for HCC patients [14]. Analyses of the samples obtained from HCC resection depicted higher expression of PD-L1, in addition to its association with tumor aggressiveness [15] and poor prognosis [16] in patients who were never treated via immunotherapy.

Blocking the interaction between PD-1 and PD-L1 leads to impressive and long-lasting anti-tumor responses in a subset of patients with many tumor types. PD-1 and PD-L1 blockades largely showed similar efficacy, though the objective response rates were 5% higher with PD-1 blockade than with PD-L1 blockade in non-small-cell lung carcinoma [17]. Agents targeting PD-1/PD-L1 have initiated a revolution also in HCC treatment as recently reviewed elsewhere [18,19].

In September 2017, anti-PD-1 antibody nivolumab (Opdivo) was approved for use by the Food and Drug Administration (FDA) for second-line treatment in sorafenib-pretreated patients with advanced HCC, based on the data derived from a dose-escalation and dose-expansion phase trial within the CheckMate-040 multi-cohort trial [20] (Table 1). The clinical activity of nivolumab was investigated in four sub-groups of advanced HCC, namely (i) sorafenib untreated or intolerant without viral hepatitis; (ii) sorafenib progressors without viral hepatitis; and (iii) HBV infected; or (iv) HCV infected HCC patients. The objective response rate was 20% in patients treated with stable dose of nivolumab and 15% in the dose-escalation phase, without differences according to the underlying liver disease [20].

Similarly, the efficacy of anti-PD-1 inhibitor pembrolizumab (Keytruda) was investigated in a phase 2 study for second-line treatment in advanced HCC patients following sorafenib failure. The study confirmed an objective response rate of 17% [21]. Thus, the FDA approved pembrolizumab for the treatment of HCC patients who have been previously treated with sorafenib in November 2018.

Despite the improvement of clinical outcomes in a subset of patients, anti-PD-1/PD-L1 blockers are still inefficient in 80% of HCC patients. Further, they are costly and cause many severe side effects [22]. There is an urgent need to define predictive factors of response to spare patients from toxicity in the absence of clinical benefits. However, none of the current trials select HCC patients according to the potential predictive factors of tumor response.

## 3. Predictive Biomarkers of Response to PD-1/PD-L1 Blockade in Order to Better Select Patients and Guide Therapeutic Choices

**Figure 1:**
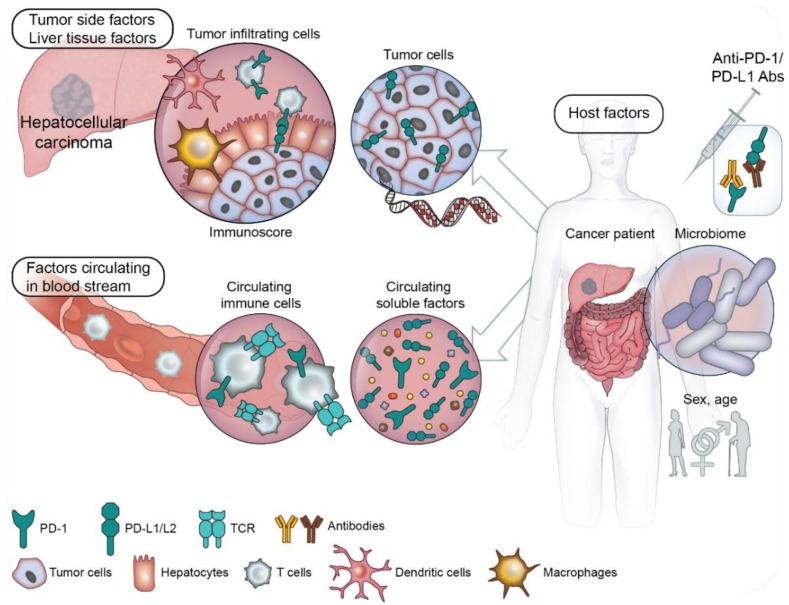
Overview of the predictive factors for PD-1/programmed cell death ligand 1 (PD-L1) blockade: potential factors to explore in HCC.

To date, very little has been described about predictive biomarkers of response to PD-1/PD-L1 blockade in HCC. Therefore, in this study, we will present predictive biomarkers highlighted in other tumor types, which could be relevant in the HCC field, in addition to recent data available for the said field. Furthermore, predictive markers of response to PD-1/PD-L1 blockade will be divided into three subsections: (i) liver tissue and tumor side factors; (ii) circulating prognostic factors; and (iii) host factors (Figure 1).

### 3.1. Liver Tissue and Tumor Side Factors

#### 3.1.1. Immunological Biomarkers

As a logical extension of our knowledge concerning the PD-1/PD-L1 pathway, the first candidate biomarkers to be explored for PD-1/PD-L1 checkpoint inhibition were immunological. Theoretically, PD-1/PD-L1 blockade should work in patients positive for PD-1 and/or PD-L1. However, we are still unravelling the complexities of the PD-1/PD-L1 interaction between tumor and different immune cell populations.

##### PD-L1 Expression by Tumor Cells and Immune Infiltrate

In 2012, it has been reported that among 17 patients with PD-L1 negative tumors, none of them responded to anti-PD-1 therapy, whereas among the 25 patients with PD-L1 positive tumors 9 presented an objective response [23]. PD-L1 expression by tumor cells was intensively studied as possible predictive biomarker for ascertaining the efficacy of anti-PD-1/PD-L1 therapy. High PD-L1 expression prior to anti-PD-1/PD-L1 therapy was later demonstrated to be associated with improved objective response rate and survival in patients with non-small-cell lung cancer [24], melanoma [25] and head and neck squamous cell carcinoma [26].

However, in patients with advanced HCC, baseline expression of PD-L1 on tumor cell did not have an impact on the objective response rates to anti-PD-1 therapy [20]. In fact, as a part of the CheckMate 040 clinical trial, tumor biopsies collected at the baseline were retrospectively assessed for PD-L1 status. Membrane expression of PD-L1 on at least 1% of the tumor cells was observed in 20% patients at the baseline and majority of patients had PD-L1 expression on less than 1% of the tumor cells. Response to therapy was observed in 26% patients with PD-L1 expression on at least 1% of the tumor cells and in 19% patients with PD-L1 on less than 1% of the tumor cells. Thus, a fraction of PD-L1-negative HCC patients showed objective clinical responses, demonstrating no significant difference compared to PD-L1 positive patients. This was recently confirmed by a study where the response to anti-PD-1 did not correlate with PD-L1 tumor staining in advanced HCC [27]. However, archival tissue samples were used and the number of evaluable patients in this study was very limited (*n* = 10).

Interestingly, PD-L1 expression on immune cells may be more predictive of anti-PD-1/PD-L1 response than PD-L1 expression on tumor cells in certain tumor types, such as bladder cancer or breast cancer [28]. This might be the case for HCC as well. In fact, the relevance of PD-L1 expression on immune cells versus that of tumor cells has been revealed by comparing three tumor models with varying sensitivity to PD-1/PD-L1 blockade. Juneja et al. demonstrated that the relative contribution of tumor-derived versus host-derived PD-L1 is context-dependent and that both these PD-L1 expressions play a role in tumor microenvironment [29]. In view of the fact that PD-L1 expression on immune cells is critical for inhibiting anti-tumor immunity, PD-L1 expression within the tumor, but not necessarily on tumor cells, may be sufficient for an anti-PD-1/PD-L1 response, as reviewed recently [30]. Thus, PD-L1 expression on immune cells should be included in the list of potential markers of response to PD-1/PD-L1 inhibition.

However, several unsolved problems remain regarding the interpretation of PD-L1 expression, such as the cut-off value to define positivity and the temporal and spatial heterogeneity of PD-L1 expression. First, the lack of standardized analyses and methods makes it difficult to compare results from individual studies, in order to reach robust overall consensus [31]. In fact, most studies evaluated PD-L1 status as the percentage of tumor cells positive for cell-surface and/or membranous PD-L1 staining. However, variable cut-off values have been used to identify positivity of PD-L1 [32]. Moreover, to detect PD-L1 staining, different types and clones of anti-PD-L1 antibodies are currently on the market. Some anti-PD-L1 antibodies result in a mixture of both membranous and cytoplasmic staining of tumor cells, which obscures the interpretation of results and affects the accuracy of the analysis [33]. Three clones of anti-PD-L1 recombinant monoclonal antibodies (Clone 28-8, 73-10, and SP142) have been approved by the FDA as complementary diagnostics for PD-1/PD-L1 checkpoint inhibitors. However, using these antibodies, some differences in detecting PD-L1 staining have still been observed [34]. Recently, five anti-PD-L1 antibody clones were used to stain HCC samples [35], showing very high diversity that impacts the reliability and reproducibility of PD-L1 assays. In addition, PD-L1 expression is inducible and can change over the course of the disease and/or during treatment [36]. Thus, the lack of standardization renders interpretation across clinical trials highly difficult.

##### Features of Intratumoral Lymphoid Infiltrates

The potential of the adaptive immune system to control or eradicate tumors has been clearly demonstrated. The immune contexture, defined by the type, location, density, and functional orientation of the tumor-infiltrating immune cells (in particular CD8^+^ cytotoxic T cells), allows one to predict the clinical outcome [37,38,39], especially in HCC [40]. Moreover, the score of immune system is a critical prognosis factor in cancer patients, and immune checkpoint blockers impact this parameter. Four different types of tumor microenvironments have been proposed by combining PD-L1 expression and T-cell density. This stratification allows one to better predict the immunotherapeutic strategy best suited to target each type [41]. Notably, different classes of HCC have been identified based on the genomic profiling of the concerned tumor microenvironment [42]. One of them, called the “immune class” (present in about 25% patients), is more susceptible to therapeutic agents blocking regulatory pathways in T cells and is characterized by markers of adaptive immune responses as well as exhausted immune responses. Therefore, it is evident that the immune contexture in HCC is critical to predict clinical outcomes following PD-1/PD-L1 checkpoint inhibition.

In addition, an IFN-γ-related signature was associated with the clinical benefits of anti-PD-1 treatment across nine different cancer cohorts [43]. In fact, the signature established from the tumor tissue at the baseline contained IFNγ-responsive genes related to antigen presentation, cytotoxic activity, chemokine expression, and adaptive immune resistance. In parallel, a resistance signature to PD-1 blockade has been identified in melanoma patients, involving high expression of the genes involved in cell adhesion, regulation of mesenchymal transition, angiogenesis, matrix remodeling, and wound healing [44]. These studies highlighted the complex biology and importance of the pre-existing tumor immune microenvironment with regard to its ability or inability to respond to PD-1/PD-L1 checkpoint inhibition.

The impact of tumor infiltration of CD8^+^ T-cell on the survival of cancer patients has been the most well-studied topic. A meta-analysis summarized that in majority of articles published, CD8^+^ immune cell infiltrates were associated with good prognosis in a wide variety of solid tumor types [45,46], and also associated with improved responses to chemotherapy and immunotherapy [47]. In addition, the number of tumor infiltrating lymphocytes expressing PD-1 was shown to be predictive of the clinical response following PD-1 blockade [48]. Similarly, it has been reported that tumor response to PD-1 blockade requires pre-existing CD8^+^ T cells that are negatively regulated by PD-1/PD-L1-mediated adaptive immune resistance [49]. Particularly, the PD-1^high^ T cells seem to be very important as this subset demonstrates higher capacity for tumor recognition and markedly different profile compared to PD-1^int^ cells in patients with non-small-cell lung cancer, where the frequency of PD-1^high^ cells strongly predicted the response and survival of patients [50]. Similarly, a clinical study performed on melanoma patients showed that PD-1^high^ expression before treatment was correlated to the response to PD-1 blockade [51].

Recently, we demonstrated that the responders to anti-PD-1/PD-L1 therapy had high baseline frequency of PD-1^high^ CD8^+^ T cells in tumor tissue, as determined by extensive phenotypic flow cytometry analyses of fresh biopsies obtained from advanced HCC patients before start of anti-PD-1/PD-L1 therapy [52]. This is in accordance with the observations of a recent study that investigated CD8^+^ T cells isolated from HCC tissue and showed in vitro that tumors with high proportions of PD-1^high^ CD8^+^ T cells are more susceptible to PD-1 blockade [13]. Similarly, high numbers of PD-1^+^ intratumoral lymphocytes predict survival benefit of cytokine-induced killer cells for HCC patients [53].

The main problem regarding the interpretation of PD-1 expression on CD8^+^ T cells is connected to the complexity of the methods needed for analyses. Simple immunohistochemistry is unable to distinguish PD-1^+^ CD8^+^ T cells since a combination of several antibodies is necessary to characterize these cells. For instance, the majority of NK cells express CD8 receptors, and their frequency is very high in the liver [54,55]. However, the CD56^bright^ subpopulation of NK cells that is present at high frequency in the liver [56] do not express PD-1 [57]. Thus, NK cells should be excluded from immunohistochemical analysis to allow correctly quantify the frequency of PD-1^+^ cells in the CD8^+^ T cell population. Moreover, tumor heterogeneity and sampling variability are inherent limitations when using liver biopsies. Due to the invasiveness of tissue sampling, only one of multiple lesions is usually selected for liver biopsy. Thus, a tissue sample might not necessarily reflect the entire picture of HCC. Additionally, both PD-1 and PD-L1 expression levels can change over time, as can the distribution of CD8^+^ T cells. Therefore, to develop clear predictive factors, specific time restrictions need to be defined, for instance, the requirement of analyzing tissue biopsies obtained at a maximum of three months prior to the start of the treatment.

In addition to tumor immune infiltrates, it is important to take into consideration that the prognostic factor for the response to PD-1/PD-L1 could also come from the non-tumoral tissue. Especially in HCC, as demonstrated previously, microarrays from surrounding non-tumoral liver tissues can predict overall survival after curative treatment of HCC, rather than the analyses obtained from tumor tissues [58]. Moreover, the frequency of infiltrated lymphocytes is much higher in a non-tumoral liver compared to a tumor area [59].

#### 3.1.2. Mutations of Tumor Cells and Microsatellite Instability

Tumor mutational burden (TMB) is a measure of the total number of mutations per coding area of a tumor genome. Tumors with higher levels of TMB are believed to express more neoantigens that may allow for a more robust immune anti-tumor response and therefore, potentially, a better response to immunotherapy. Certainly, high TMB and neoantigen load have been noted to predict the response to immunotherapies, including anti-PD-1 therapy (higher objective response rate and/or prolonged survival) in melanoma, non-small-cell lung carcinoma [23], and across diverse tumors [60]. When compared to other tumor types, HCC is described by an above-average TMB with frequent formation of neoantigens [61], expected to have a good response to PD-1/PD-L1 blockage. Nevertheless, TMB is a rough marker because a mutation could or could not be immunogenic. Currently, bioinformatics tools are available to better predict the immunogenicity of mutations [62]. Recently, next-generation sequencing recognized Wnt/CTNNB1 mutations, typical for the immune-excluded tumor class, as possible biomarkers predicting resistance to immune checkpoint inhibitors in patients with advanced HCC [63]. However, this type of sequencing is complex and costly, therefore difficult for routine clinical use. Microsatellite instability (MSI) is a phenotype of hyper-mutations arising from mismatch-repair deficiency (dMMR), that is the first predictive biomarker for anti-PD-1 blockage approved by the FDA [64]. To be more precise, in May 2017, the FDA granted accelerated approval to pembrolizumab for pediatric and adult patients suffering from unresectable or metastatic MSI or dMMR solid tumors that have progressed following first-line treatment, in addition to the standard of care. Previously, MSI-high tumors were observed to display upregulation of multiple immune checkpoints, including PD-1, thus making PD-1/PD-L1 blockade a rational treatment approach. In an expanded study of advanced dMMR cancers across 12 different tumor types, objective radiographic responses were observed in 53% of patients, while complete responses were achieved in 21% of patients across 12 different tumor types [65]. However, in HCC, MSI seems to be a rare event [66].

### 3.2. Circulating Prognostic Factors

Circulating markers possess the advantage of being suitable for sampling over the course of treatment period and may be quickly established and accessible for clinical practice.

#### 3.2.1. Circulating Immune Cells

The predictive value of circulating markers has been evaluated in melanoma patients treated with pembrolizumab. Moreover, high relative eosinophil and lymphocyte count were associated with favorable overall survival [67]. In another study, high relative eosinophils and basophils, low absolute monocytes, and a low neutrophil-to-lymphocyte ratio served as significant independent variables for favorable overall survival of patients with advanced melanoma [68]. Additionally, T-cell receptor (TCR) diversity could be a critical determinant of the clinical outcome regarding PD-1/PD-L1 checkpoint inhibition. A high pre-treatment clonality of TCR (indicative of a repertoire that is not diverse) was associated with poor clinical outcomes in patients with urothelial cancer treated with anti-PD-L1 [69].

In HCC, the expression of immune checkpoint molecules, such as PD-1, Tim-3, and Lag-3, in the tumor tissue may be partially reflected on the circulating immune cells [12,13,52], and immunomonitoring conducted at the circulating level has the potential to highlight prognosis factors of clinical evolution and distinguish responders from non-responders.

#### 3.2.2. Circulating Soluble Factors

Recently, Feun et al. published a study where high baseline plasma levels of anti-inflammatory cytokine TGF-β were significantly correlated with poor outcomes after anti-PD-1 treatment in patients with advanced, unresectable HCC [27]. This promising finding is based on small cohort of patients and requires a larger number of patients for confirmation.

A baseline protein signature of patients that were PD-1 resistant as analyzed via mass spectrometry, was characterized by complement, acute phase, and wound healing molecules in metastatic melanoma patients receiving PD-1 blocking antibody [70]. Soluble immune checkpoints may also serve as potent biomarkers of response to PD-1/PD-L1 checkpoint inhibition, as it has been shown that elevated pre-treatment levels of soluble PD-L1 were associated with a progression in melanoma patients treated with PD-1 blockade [71]. In a cohort of HCC patients, the high level of the soluble PD-L1 was correlated with a poor outcome [72] but the association between soluble immune checkpoints and the response to PD-1/PD-L1 needs to be further investigated.

Extracellular vesicles such as exosomes and microvesicles are actively released from various cells, including cancer cells, and carry bioactive molecules that influence the immune system. A recent study from Chen et al. indicated that the circulating exosomal PD-L1 may reflect the states of anti-tumor immunity in melanoma patients as responders to anti-PD-1 were characterized by the increase in circulating exosomal PD-L1 during early stages of treatment [73]. However, the application of exosomal PD-L1 as a possible predictor for anti-PD-1 therapy remains controversial. HCC-derived exosomes and their potential as biomarkers were recently reviewed elsewhere [74].

### 3.3. Host Factors

#### 3.3.1. Sex and Age

Recently, Conforti et al. provided evidence for the fact that the benefit of immune checkpoint inhibitors might be sex dependent [75]. In their meta-analysis of 20 randomized controlled trials testing anti-PD-1 and anti-CTLA-4, the authors observed a significantly higher overall survival benefit for men than women. This could be predictable if we consider that on an average, women mount stronger immune responses than men, and this immune response is hypothesized to lower their risk of cancer-related mortality [76,77]. Men are at almost two-times higher risk of mortality from most cancers, including HCC [78], compared to women. This male-biased mortality reflects differences not only in behavioral and biological factors but also in the immune system that is less active in men, including less effective anti-tumor immune responses [76,77]. Therefore, men’s immune system might be easier to activate via immunotherapies targeting immune cells. Sex hormones influence innate and adaptive immune responses [76,77,79] and directly regulate the expression as well as function of PD-1 and PD-L1 [80,81]. A retrospective analysis found that the female sex and the age <65 years are associated with lower objective response rates to anti-PD-1 therapy compared to the male sex [82]. Thus, as far as sex hormones are concerned, it should be noted that aging is associated with the loss of sex hormones in both men and women. Thus, sex-dependent differences might disappear in part with age. In addition, the immune system is less active in older patients. As anti-PD-1/PD-L1 antibodies are therapies that should restore a lost anti-tumor immunity [83], older patients may benefit more from this treatment. Recently, Kugel et al. reported that patients aged over 60 had better response to anti-PD-1 therapy, and the likelihood of response increased with age [84]. Nevertheless, further studies are required in this regard to provide clues pertaining to the effectiveness of immunotherapy according to one’s sex and age among HCC patients.

#### 3.3.2. Influence of the Gut Microbiome

Recent evidence suggests that modulation of the gut microbiome may affect responses to immunotherapy. In fact, a significant association was observed between commensal microbial composition and clinical response to PD-1/PD-L1 therapy in melanoma patients [85]. Moreover, extensive work on the biology of the gut–liver axis has assisted in better understanding the relationship between the said microbiome and HCC [86]. For instance, in patients suffering from cirrhosis and fatty liver, the gut microbiota profile and systemic inflammation were significantly correlated and linked to HCC development [87]. A recent review summarized the knowledge about the modulatory effect of gut microbiota on immune system leading to chronic inflammation and HCC development [88]. Additionally, Zheng et al. reported the characteristics of the gut microbiome during anti-PD-1 immunotherapy in HCC, by metagenomic sequencing of periodic fecal samples [89]. Authors observed that fecal samples from patients responding to immunotherapy (*n* = 3) showed higher taxa richness and more gene counts compared to non-responders (*n* = 5), suggesting for the first time that gut microbiome may affect the response to anti-PD-1/PD-L1 immunotherapy in patients with HCC. Thus, the role of the gut microbiome in response to PD-1/PD-L1 checkpoint inhibition in HCC patients needs to be further investigated.

## 4. Conclusions

Although PD-1/PD-L1 checkpoint inhibition has improved the response rate for HCC, such treatments help only a minority of patients at present. A major focus involves determining the reason immunotherapies succeed or fail, in addition to the way they can be improved further. Predictive biomarkers are necessary to identify HCC patients with a greater likelihood of response, thereby guiding clinical decision-making for first-line and second-line therapies. However, even the most promising predictors of response to anti-PD-1/PD-L1 therapy in HCC, low baseline plasma levels of TGF-β or high frequency of intratumoral CD8^+^ or PD-1^high^ CD8^+^ T cells, need to be verified using a larger number of patients in a prospective trial. Thus, in order to propose a clinical decision-making algorithm in HCC based on such biomarkers, extensive translation research is currently required.

## Figures and Tables

**Table 1:** Results obtained from clinical trials of programmed cell death protein 1 (PD-1) inhibitors in hepatocellular carcinoma (HCC). Administration every two weeks (Q2W) and every three weeks (Q3W).

Agent (Clinical Trial)	Dose	Objective Response Rate	Partial Response	Complete Response	Reference
Nivolumab (CheckMate 040)	Escalation 0.1–10 mg/kg (Q2W)	15%	4/48 (8.3%)	2/48 (4.2%)	[20]
Nivolumab (CheckMate 040)	Expansion 3 mg/kg (Q2W)	20%	39/214 (18.2%)	3/214 (1.4%)	[20]
Pembrolizumab (KEYNOTE-224)	200 mg (Q3W)	17%	17/104 (16%)	1/104 (1%)	[21]
